# Analysis of the subcapital two-part humerus fracture by fluoroscopy: objective criteria for classification and decision making

**DOI:** 10.1007/s00402-021-03940-x

**Published:** 2021-06-02

**Authors:** Nicolas Bless, Nicola Keller, Amir Steinitz, Thibaut Klein, Daniel Rikli

**Affiliations:** 1 Department of Orthopedics and Traumatology, University Hospital Basel, Spitalstrasse 21, 4031 Basel, Switzerland; 2 University of Basel, Basel, Switzerland; 3 Clinic for Orthopedics and Surgery, Merian Iselin, Basel, Switzerland; 4 Department of Biomedicine, University of Basel, Basel, Switzerland

**Keywords:** Shoulder, Proximal humerus fracture, Angulation, Surgical neck, Fluoroscopy, Classification

## Abstract

**Background:**

Surgical decision making in the treatment of proximal humerus fractures (PHFx) is primarily based on fracture classification using standard radiographs. Due to the lack of objective criteria, this classification process is associated with high interobserver variation. In this study, we investigate the fluoroscopic analysis of humerus fractures through the surgical neck using a semi-quantitative determination of distinct angulation patterns of the proximal humerus as they appear in the image intensifier.

**Methods:**

Using a saw bone model, defined subcapital 2-part fracture configurations were generated and assessed radiographically. Anatomical landmarks—including the greater and lesser tuberosity as well as anatomical neck—were identified using an image converter, and the exact degree of fracture displacement with 10° up to 70° (in 10° increments) of posterior, varus or combined posterior-varus angulation was compared to nondisplaced controls. From the resultant series of radiographs, the appearance of these angulations in anteroposterior (AP) and scapular Y-views were also visualized and defined.

**Results:**

An angulation of 50° or more of any given 2-part fracture through the surgical neck is present when the greater tuberosity becomes the most proximal point in AP view (varus and combined posterior-varus angulation) or a bimodal form is found for the superior contour of the head with the lesser tuberosity being the most proximal point in the Y-view (posterior angulation).

**Conclusion:**

The radiological appearance of various PHFx constellations can be well visualized using the saw bone shoulder model. The presence of angulation in accordance with the Neer classification for group III fractures can be adequately determined by analyzing the relative position of the greater or lesser tuberosity to the humeral head calotte. This can assist the surgeon’s decision on whether to operate or opt for a conservative approach.

**Level of evidence:**

Basic Science, Anatomy Study, Imaging.

## Introduction

Proximal humerus fractures (PHFx) are the third most common bone fractures worldwide, accounting for 6% of all fractures [1]. With the marked rise in incidence due to the aging population [2, 3], PHFx plays a prominent role in trauma surgery. Nonoperative treatment of PHFx is considered routine regardless of patient age [4]. The decision for surgery, on the other hand, is influenced not only by patient age but by the presence of associated orthopedic injuries, fracture severity and the presence of associated glenohumeral dislocation [5].

Due to the extensive fracture combinations that can occur, PHFx classification can be challenging [6], even with the availability of the well-established AO and Neer systems [7, 8]. The latter system distinguishes all minimal displaced fractures as ‘1-part fractures' because they are viewed as a stable unit and therefore, can be conservatively treated. In contrast, fractures through the surgical neck are classified as displaced either by the displacement of the shaft by more than 1 cm or an angulation of the head of more than 45° [8]; without further guidelines the degree of angulation is difficult to recognize on a standard radiograph. Interobserver reliability for the classification of PHFx is generally low and was demonstrated as only “fair” especially for the assessment of head-shaft angulation in the coronal and sagittal plane [9]. Furthermore, most surgery indications are based on the physician’s opinion regarding fracture severity, which adds to the lack of objectivity in planning the right treatment for PHFx [5, 10, 11].

Fluoroscopy provides an immediate sequence of radiographic images, which allows the surgeon to intraoperatively evaluate static or dynamic pictures of the structure of interest. Therefore, our goal was to investigate the fluoroscopic analysis of humerus fractures through the surgical neck using a semiquantitative determination of distinct angulation patterns of the proximal humerus. With this strategy, we developed guidelines for the interpretation of how distinct 2-part surgical neck angulation patterns of the proximal humerus present themselves in standard views of conventional radiographs and under image intensifier.

## Materials and methods

**Fig. 1:**
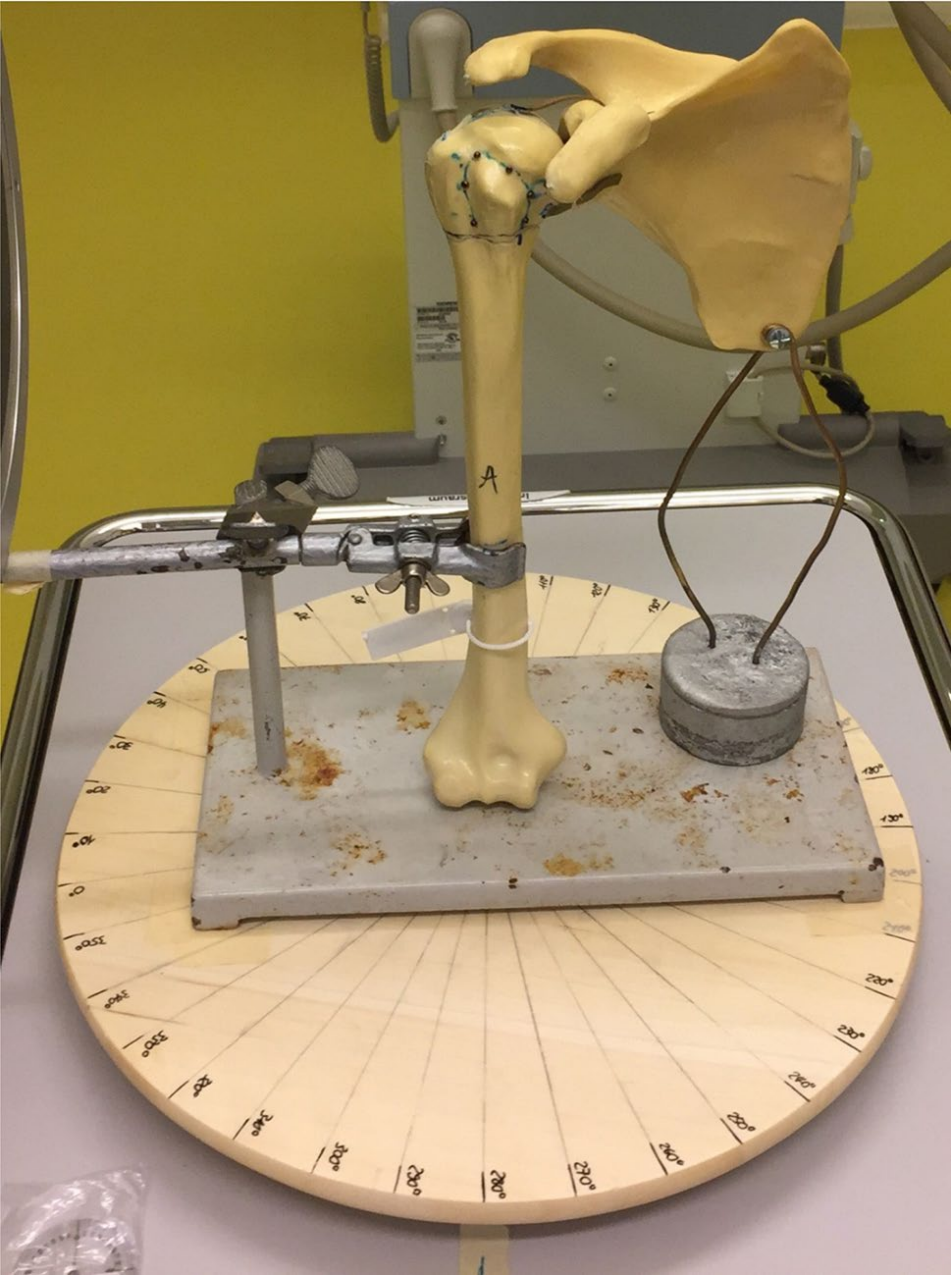
Setup of the saw bone shoulder with defined anatomical landmarks and positioned on the turntable with 10° increments within the 360° range

For this PHFx morphology study, we used a Sawbones plastic shoulder joint phantom (Pacific Research Laboratories Inc., Vashon, WA). Eleven silver marker beads (Rocailles, Rayher Hobby GmbH, Laupheim, Germany) with a diameter of 2 mm were glued on the shoulder joint, five of which were used to mark the greater tuberosity, four the glenoid and two the distance between the acromion and coracoid process. The phantom humerus was then fixed to a 16 cm long burette clamp attached to a regular support stand. This construct was placed on a wooden turntable with a diameter of 39 cm where every 10° angle was marked (Fig. 1). The scapula of the shoulder joint phantom was fixed to a separate construct; a screw was drilled into the inferior angle and held with two adjustable copper wires fixed into a metal weight. This arrangement was created to ensure the scapula could stand alone without the humeral head (where localized fractures would be generated), but be placed in the correct anatomical position by an experienced orthopedic surgeon. The position was noted with a waterproof marker to consistently replicate the same setting.

The radiographic procedure was done with an Arcadis Varic mobile fluoroscopic system (Siemens AG, Munich, Germany). Our saw bone model was positioned between the *x*-ray tube and image intensifier. Radiographs were taken and all anatomical landmarks were documented with the mobile C-arm fluoroscopy unit (39 cm source-to-image distance, 1024 × 1024 image resolution, DICOM format). To characterize humeral bone dynamics within the range of 360°, the turntable was rotated stepwise by 10° each time to link each view with the specific constellation of the marked anatomical key structures. For the comparison of different angulations between the humerus head and humerus shaft, only true AP and scapular Y-views were applied.

### OsiriX DICOM viewer

**Fig. 2:**
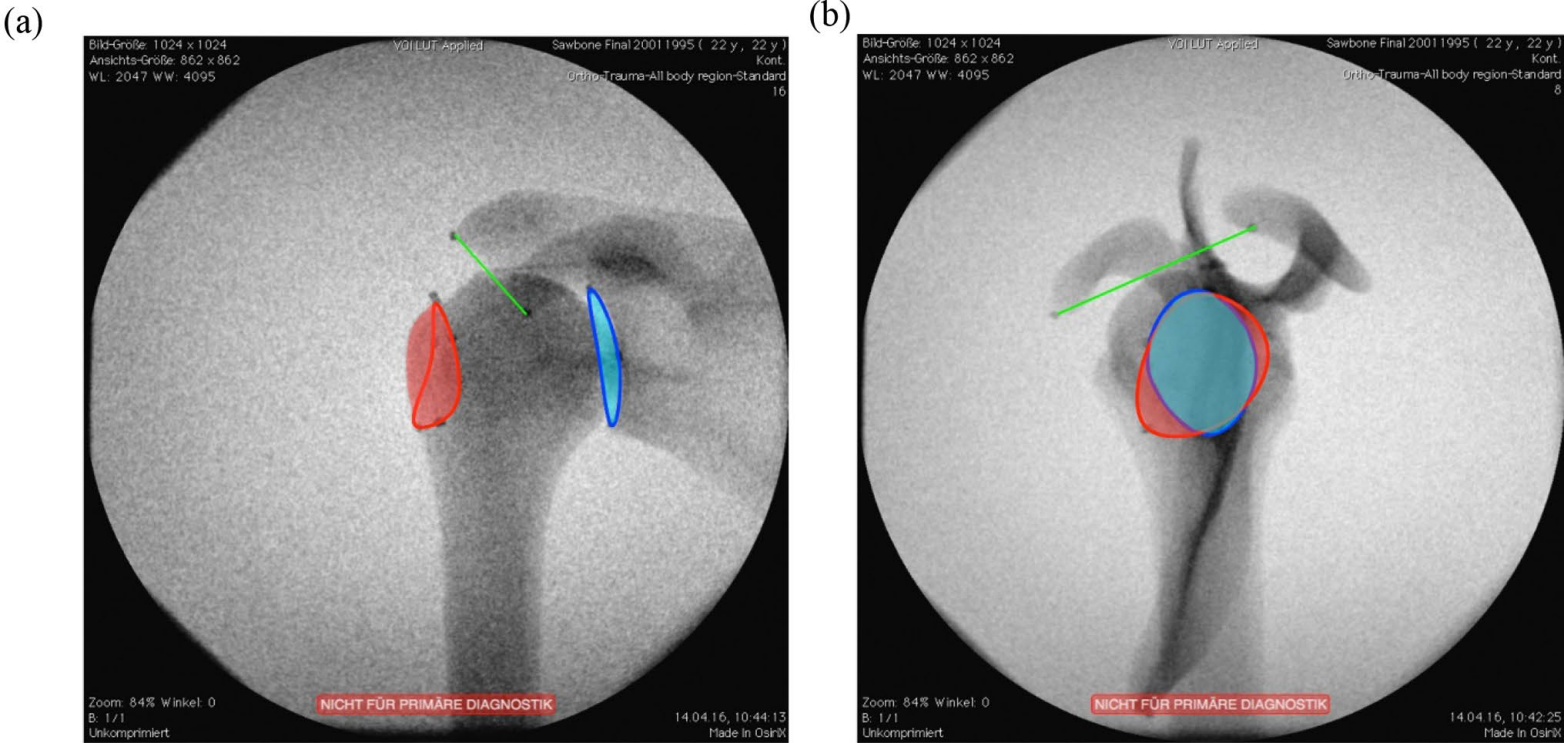
Two of the 36 radiographs of the saw bone shoulder: **a** anteroposterior and **b** scapular Y-views showing the greater tuberosity (red), glenoid (blue) and distance between the acromion and coracoid process (green)

DICOM images were viewed and marked using OsiriX Version 10.0.2 (Pixmeo SARL, Bernex, Switzerland) and then edited in Adobe Photoshop^®^ CS6 Version 13.0.4 × 64 (Adobe Inc., San José, CA) to improve landmark visibility as well as link the resultant 36 images with a GIF file (Fig. 2). The markings were done in OsiriX using the polygon tool that connects the marker beads with an outline, and the filling of the area was done in Adobe Photoshop^®^ CS6 with the standard brush function.

### Artificial fracturing

**Fig. 3:**
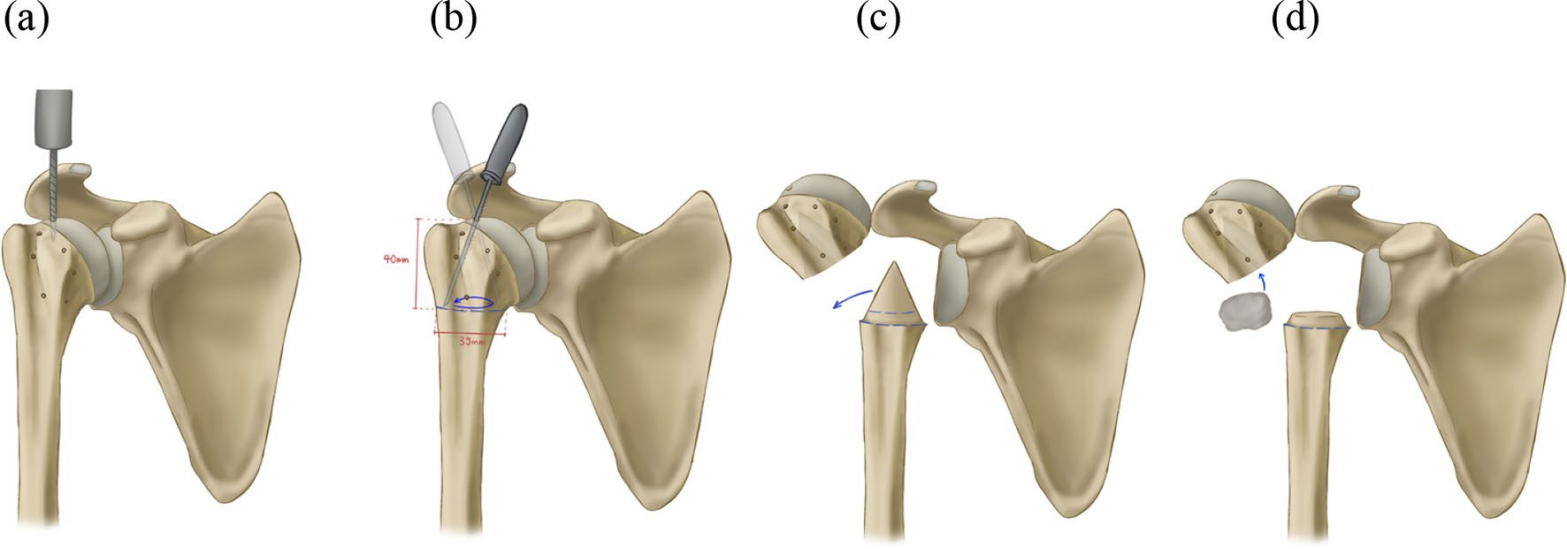
Preparation of a 2-part fracture through the surgical neck in the saw bone shoulder. **a** A hole at the top of the humeral head was drilled, **b** a circumferential cut was made 40 mm below the tip of the calotte and **c** a cone-shaped piece of the plastic saw bone was cut out to ensure head mobility. Putty was finally applied to fill the free space of the missing cone (**d**)

**Fig. 4:**
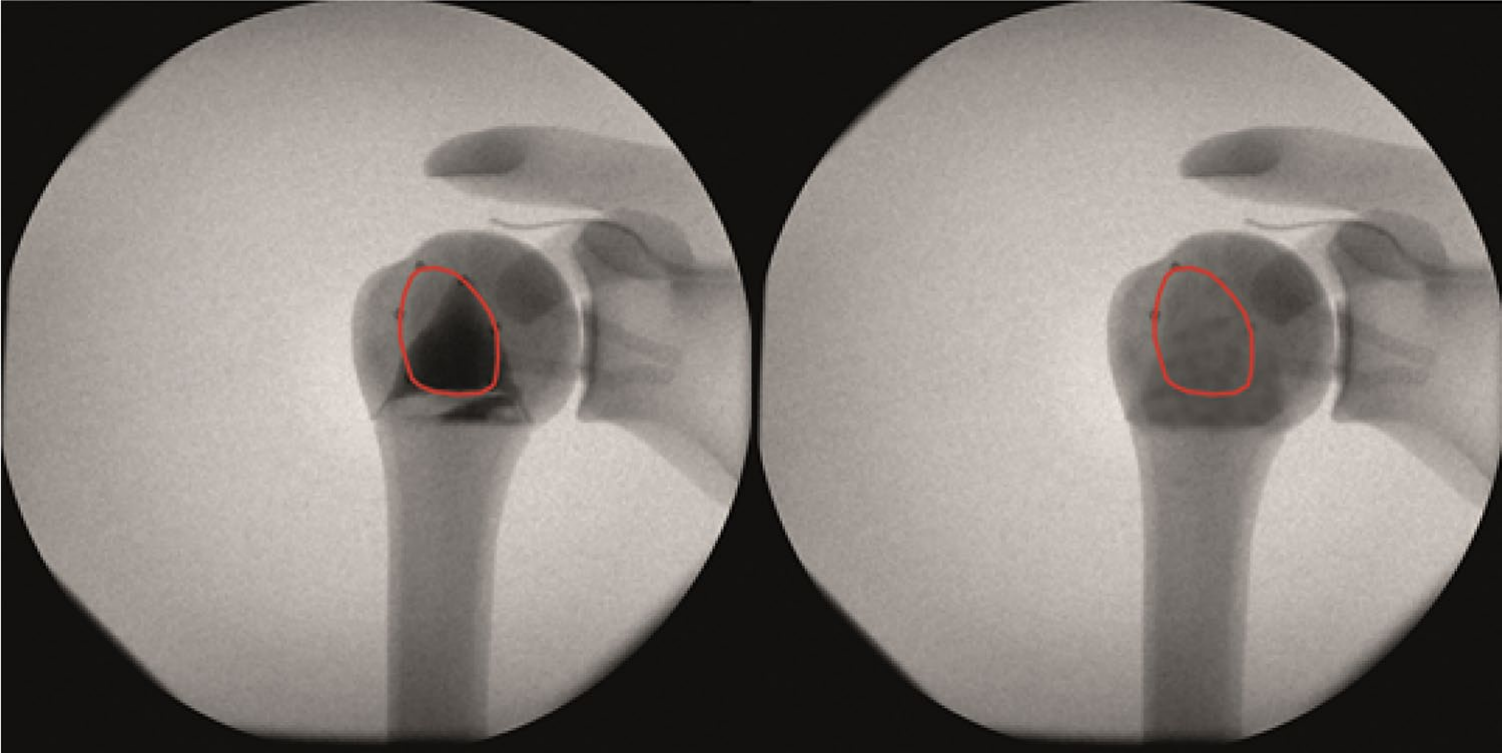
Anteroposterior view radiograph of the saw bone shoulder prepared with the 2-part fracture before (left) and after (right) image editing

A 2-part fracture through the surgical neck (equivalent to Neer group III) was manually generated in the surgical neck with a hand saw. The fracture was located 4 cm distal from the most proximal point on the calotte. A hole at the top of the humerus head was drilled, and a cone-shaped piece of the plastic saw bone was cut out to ensure head mobility (Fig. 3). The free space from the missing cone was filled with Creall^®^ Super Soft modelling putty (Havo BV, Ermelo, Netherlands) to fix the humerus head on the shaft and enable neck-shaft angle adjustments up to 70° displacement without the head falling off. On radiographs, the putty was made less visible using the stamp brush function of Adobe Photoshop^®^ CS6 as well as area patterns outside of the putty area without altering the original depiction of the landmarks (Fig. 4).

### Radiograph series

Radiographs were taken in the anteroposterior (AP) and scapular Y-views. True AP and scapular Y-views were ensured by rotating the wooden turntable until the optimal position between the x-ray tube and image intensifier was achieved. The 10° angulation marks on the wooden turntable were helpful for replication of the exact same setup between tests. True AP projections of the humerus were achieved by aiming the *x*-ray beam at a right angle to a line running through the epicondyles, and by positioning the humeral shaft in a vertical direction. True scapular Y-views were accomplished by aiming the *x*-ray perpendicular to the scapular body. Fracture displacements of 10°, 20°, 30°, 40°, 50°, 60° and 70° of posterior and/or varus angulation for each landmark were assessed. The combined posterior-varus angulation was measured with the same angle in both directions. Angles were measured with a plastic goniometer.

A total of 88 images were taken in two series: 44 images with markings on the greater tuberosity and 44 images with markings on the lesser tuberosity. Each series consisted of two images in the neutral position as well as 14 images each in the posterior, varus and combined posterior-varus position, respectively.

For every image, color markings were added to indicate various anatomical areas and lines. Red areas represent the greater or lesser tuberosity, and blue lines show the proximal contour of the humeral head. Two additional horizontal lines were drawn to mark the most proximal point on the head contour in blue, and the most proximal point of the greater or lesser tuberosity in red.

## Results

**Fig. 5:**
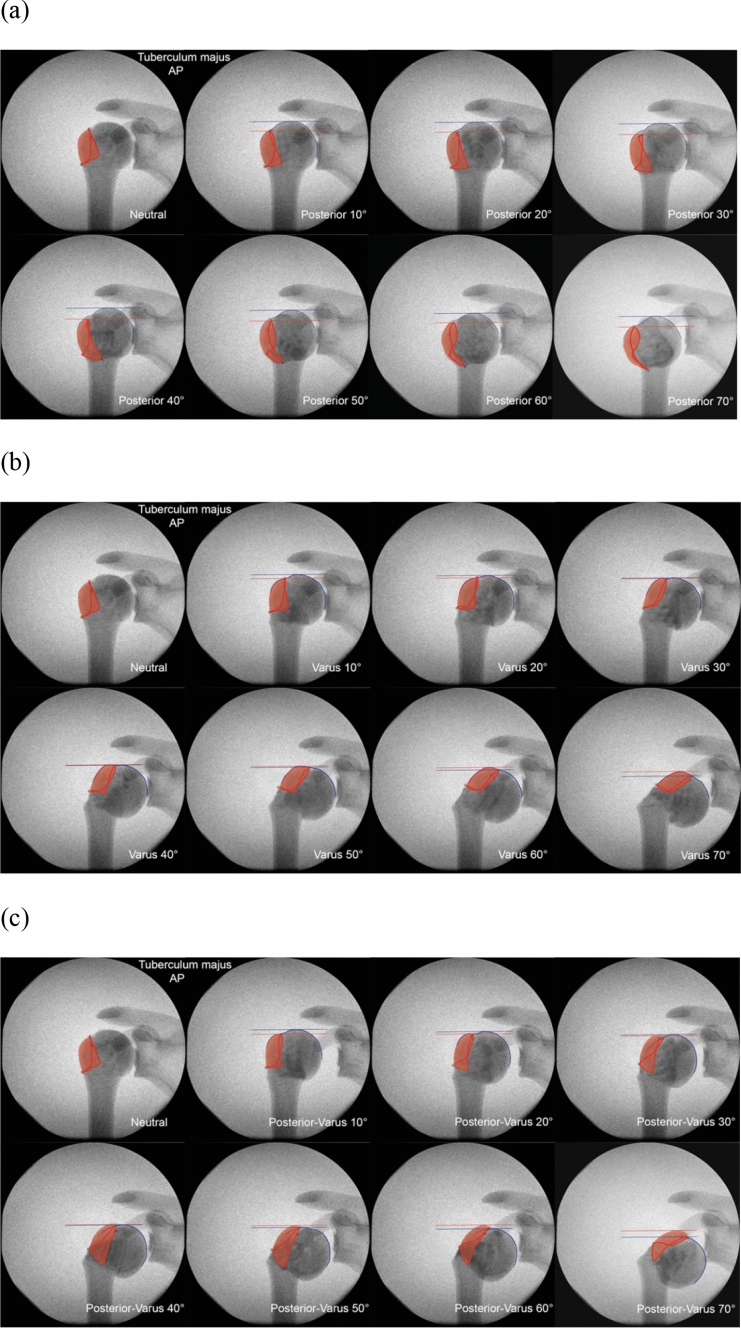
Anteroposterior view radiographic series of the greater tuberosity (red) with increasing **a** posterior, **b** varus, and **c** combined posterior-varus angulation. Blue and red lines indicate the most proximal point on the head contour and tuberosity, respectively

**Fig. 6:**
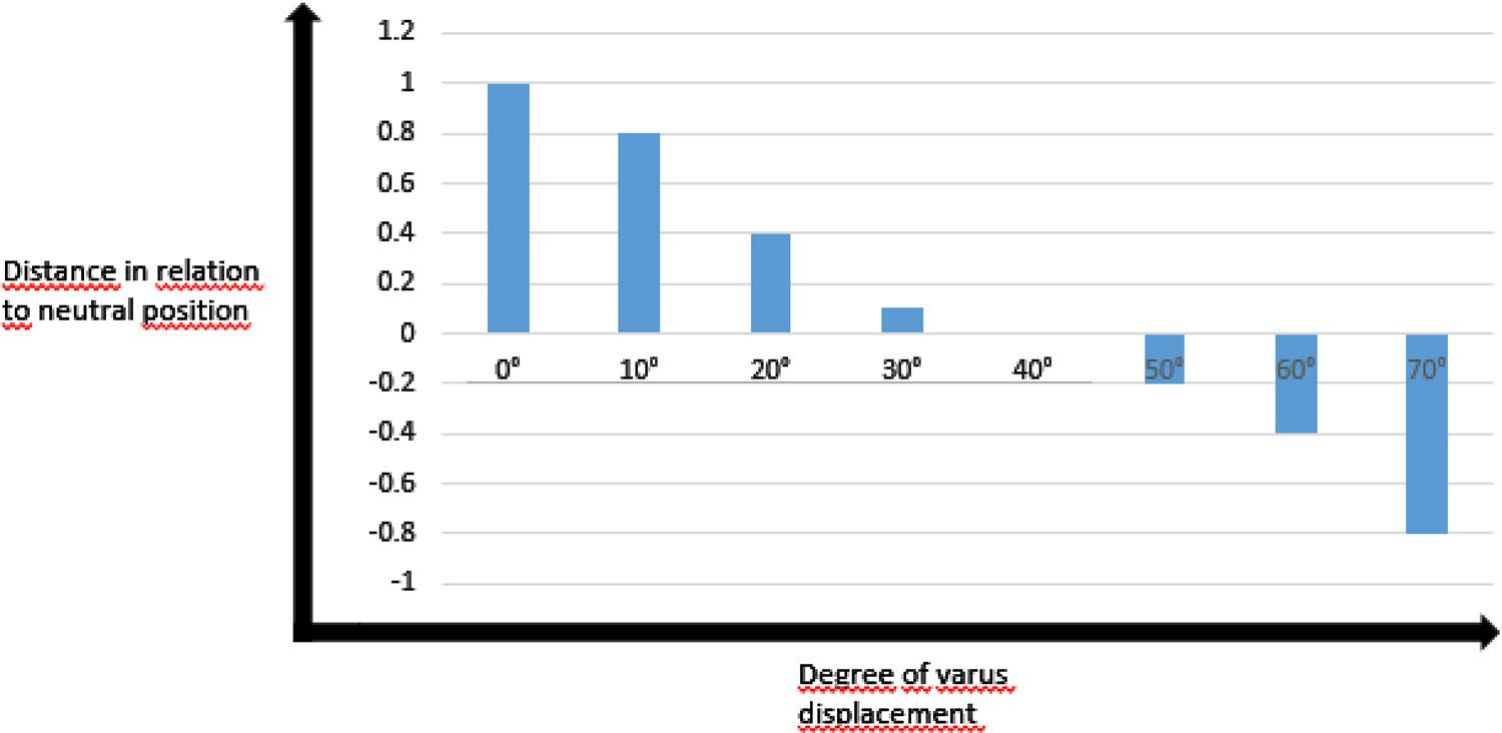
Relative distance between the most proximal point of the greater tuberosity and the tip of the humeral calotte versus varus displacement in the anteroposterior view

Increasing the posterior angulation of the head did not change its lateral contour (Fig. 5a); the degree of posterior angulation could not be judged based on the position of the greater tuberosity. Increasing varus angulation, however, resulted in a rise of the greater tuberosity relative to the cranial tip of the calotte (Fig. 5b). When the greater tuberosity rose above a horizontal line drawn perpendicular to the humeral shaft at the height of the cranial tip of the calotte, angulation was close to 50° and at this point, clearly fulfills the Neer criteria for group III PHFx (Fig. 6). The same rule applies to the combined posterior-varus angulation constellation (Fig. 5c).

**Fig. 7:**
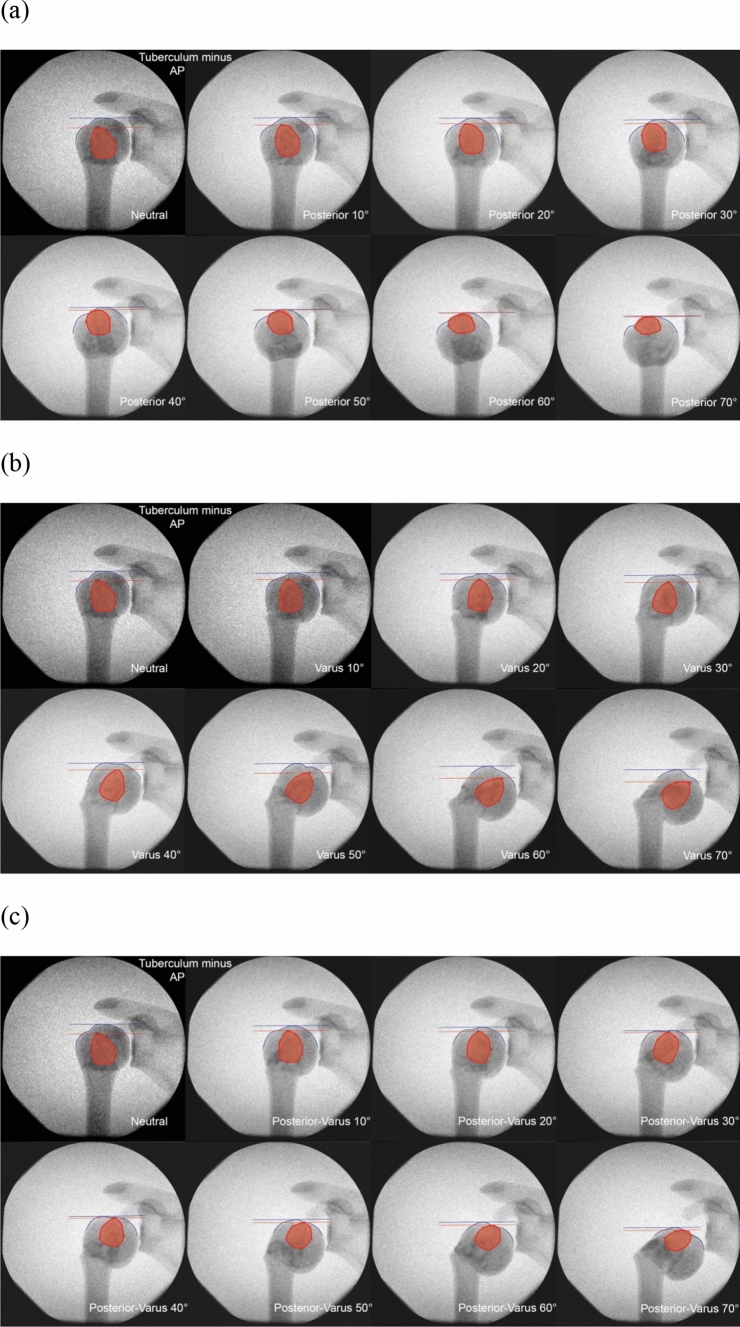
Anteroposterior view radiographic series of the lesser tuberosity (red) with increasing **a** posterior, **b** varus, and **c** combined posterior-varus angulation. Blue and red lines indicate the most proximal point on the head contour and tuberosity, respectively

Although the appearance of the lesser tuberosity—when visually enhanced with the applied markers—did change to some extent, increasing the angulation in any of the tested directions did not have any notable effect on the outer contour of the head, which hindered the assessment of dislocation (Fig. 7).

**Fig. 8:**
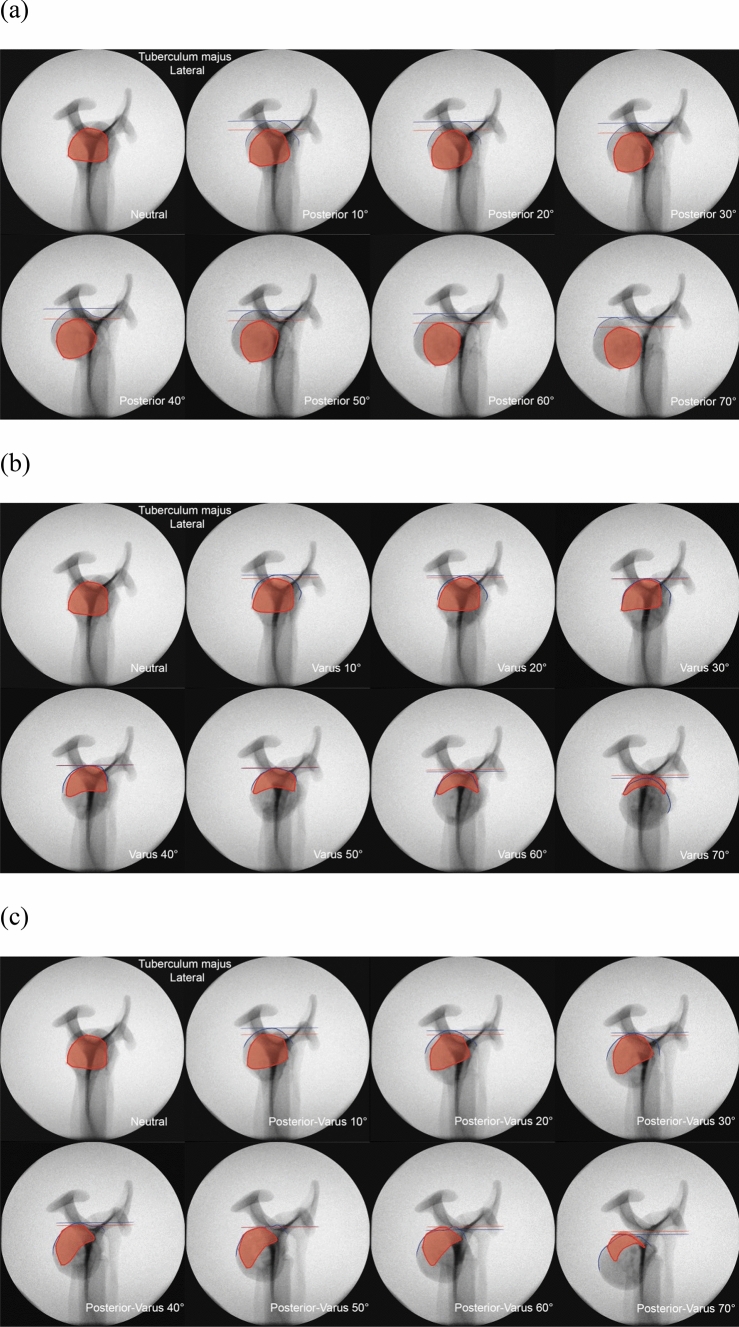
Scapular *Y*-view radiographic series of the greater tuberosity (red) with increasing **a** posterior, **b** varus, and **c** combined posterior-varus angulation. Blue and red lines indicate the most proximal point on the head contour and tuberosity, respectively

In a similar manner to that of the lesser tuberosity, the appearance of the greater tuberosity did clearly change in the lateral view when visually enhanced with the applied markers (Fig. 8). Increasing the angulation in posterior, varus or combined directions also had little effect on the outer contour of the head.

**Fig. 9:**
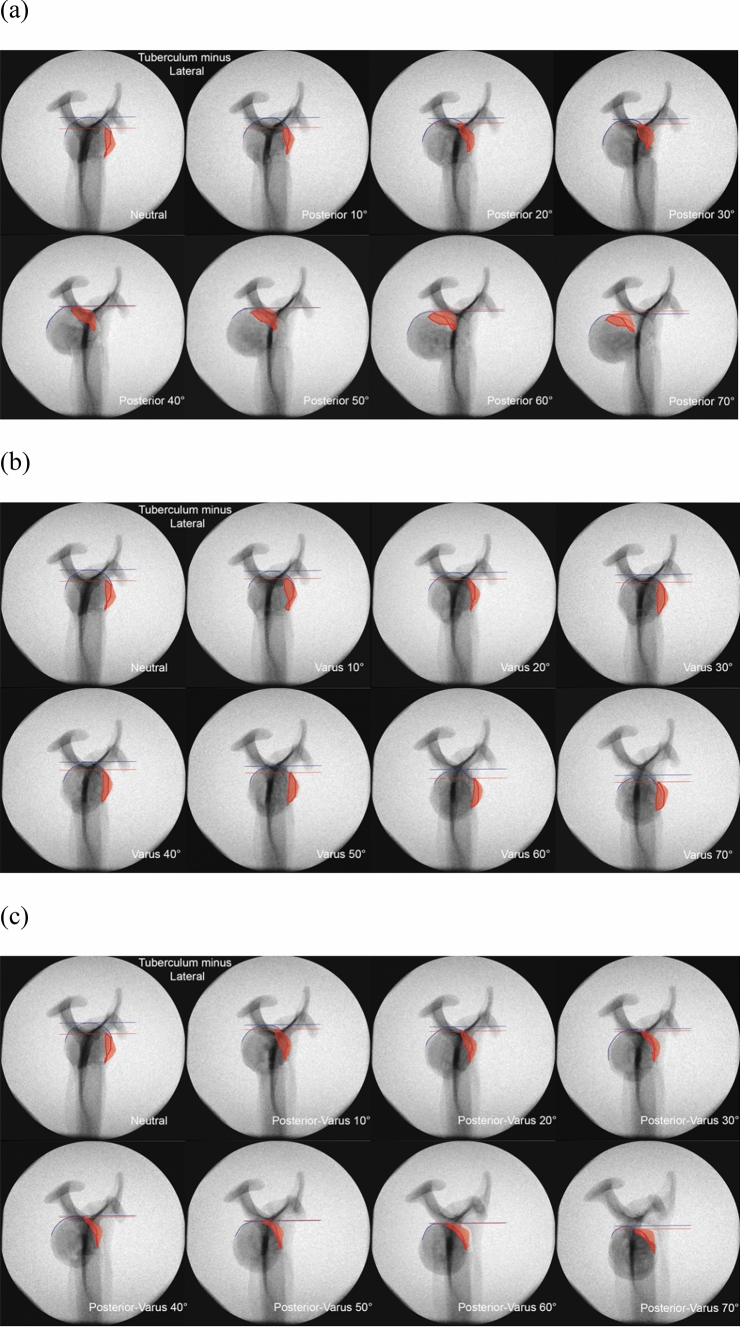
Scapular *Y*-view radiographic series of the lesser tuberosity (red) with increasing **a** posterior, **b** varus, and **c** combined posterior-varus angulation. Blue and red lines indicate the most proximal point on the head contour and tuberosity, respectively

By increasing posterior angulation, there was a rise of the lesser tuberosity relative to the cranial tip of the calotte (Fig. 9a). When the lesser tuberosity rose above a horizontal line drawn perpendicular to the humeral shaft at the height of the cranial tip of the calotte, this resulted in a bimodal contour of the head. The angulation was close to 50°, which fulfills the Neer criteria for group III PHFx. Increasing varus angulation did not change the anterior contour of the head (Fig. 9b); the degree of varus angulation could not be judged based of the position of the lesser tuberosity. With combined posterior-varus angulation, the lesser tuberosity rose in a similar manner to that observed with posterior angulation, yet did not rise above a horizontal line drawn perpendicular to the humeral shaft at the height of the cranial tip of the calotte (Fig. 9c).

## Discussion

The most important finding of this study was that 2-part PHFx through the surgical neck can be categorized in terms of angulation according to the Neer classification. Existing classifications for PHFx are limited by the difficulty to assess the extent of angulation on conventional radiographs and under fluoroscopy. In the current study, we present distinct criteria for the assessment of angulation in 2-part fractures through the surgical neck in two standard views of conventional radiographs and under image intensifier. This method is effective in analyzing both the fracture pattern and quality of fracture reduction as well as implant positioning [12]. While the direction of dislocation is easily assessed by fluoroscopy, there is a lack of reliable criteria to establish the extent of angulation. Accurate assessment of the bone morphology and correct analysis of the fracture are ultimately essential for good functional outcome [13–15].

An angulation of 50° or more for any given 2-part fracture through the surgical neck is found when the greater tuberosity becomes the most proximal point on an AP view image (varus and combined posterior-varus angulation) or a bimodal form is found for the superior contour of the head with the lesser tuberosity being the most proximal point on a scapular *Y*-view image (posterior angulation). Having distinct criteria for the assessment of angulation is a precondition for validating clinical studies focused on assessing treatment outcomes for Neer group III fractures.

Gracitelli and coworkers demonstrated that the determination of the presence of fracture and displacement of the greater tuberosity and medial metaphyseal comminution is reliable with the use of simple radiographs [9]. The interobserver reliability for head angulation in both coronal and sagittal planes was nevertheless considered to be only “fair” [9]. A dependable method to assess the head-shaft angle under fluoroscopy or on conventional *x*-ray images should therefore be especially helpful to improve interobserver reliability. The known limitations of the Neer classification system in terms of outcome prediction may also be improved by a better understanding of fracture morphology as shown by Fisher et al. [16].

Several limitations need to be addressed. First, due to the in vitro nature of our work that used a saw bone model without the presence of intact soft tissue, the digital images analyzed might have a better quality compared to in vivo images. All pictures were taken from a single model, which cannot account for the variation in size and shape of the humeral head for every patient. Second, the images were edited to enhance the clarity of bone structures. While the anatomical borders of the greater and lesser tuberosity are less visible on patient images, the contour of the head and critical anatomical landmarks remain well recognized. The results of this study are strictly limited to 2-part fractures through the surgical neck; when the fracture pattern implies, for example, that the greater or lesser tuberosity is no longer in its correct anatomical position in relation to the rest of the calotte, the rules as defined in our study no longer apply. Finally, a number of technical limitations must also be noted: The test set-up was put together several times and although this was always done in a standardized manner, minimal differences between each set-up may exist. Although the angles were manually measured with a standard analog goniometer, a more accurate technique with an increased measurement range of combined posterior-varus angulation could have been employed. Markers were glued around the greater and lesser tuberosity using only five to six beads each; the connection of these points was highlighted using a specific computer tool that cannot ensure 100% accuracy.

## Conclusion

The presence of an angulation in accordance with the Neer classification for group III fractures is adequately determined by analyzing the relative position of the greater or lesser tuberosity to the humeral head calotte. This method can assist the surgeon’s decision on whether to operate or choose a conservative approach. With the presented criteria, a higher interrater reliability for the Neer classification can be expected. Improved differentiation between Neer group I and Neer group III fractures will be helpful for any validation study using this classification.
